# Genetic background and immunological status influence B cell repertoire diversity in mice

**DOI:** 10.1038/s41598-019-50714-y

**Published:** 2019-10-03

**Authors:** Nancy Chaaya, Melody A. Shahsavarian, Irene Maffucci, Alain Friboulet, Bernard Offmann, Jean-Benoist Léger, Sylvain Rousseau, Bérangère Avalle, Séverine Padiolleau-Lefèvre

**Affiliations:** 1 CNRS UMR 7025, Génie Enzymatique et Cellulaire. Centre de Recherche de Royallieu. CS 60319, 60203 Compiègne Cedex, France; 2 Sorbonne Universités, Université de Technologie de Compiègne, Génie Enzymatique et Cellulaire. Centre de Recherche de Royallieu. CS 60319, 60203 Compiègne Cedex, France; 3 Université de Nantes, Unité Fonctionnalité et Ingénierie des Protéines (UFIP), UMR 6286 CNRS, UFR Sciences et Techniques, 2, chemin de la Houssinière, 44322 Nantes, France; 4 CNRS UMR 7253, Heudiasyc; Université de Technologie de Compiègne. Centre de Recherche de Royallieu. CS 60319, 60203 Compiègne Cedex, France; 5 Sorbonne Universités, Université de Technologie de Compiègne, Heudiasyc. Centre de Recherche de Royallieu. CS 60319, 60203 Compiègne Cedex, France

**Keywords:** Autoimmunity, Immunogenetics

## Abstract

The relationship between the immune repertoire and the physiopathological status of individuals is essential to apprehend the genesis and the evolution of numerous pathologies. Nevertheless, the methodological approaches to understand these complex interactions are challenging. We performed a study evaluating the diversity harbored by different immune repertoires as a function of their physiopathological status. In this study, we base our analysis on a murine scFv library previously described and representing four different immune repertoires: i) healthy and naïve, ii) healthy and immunized, iii) autoimmune prone and naïve, and iv) autoimmune prone and immunized. This library, 2.6 × 10^9^ in size, is submitted to high throughput sequencing (Next Generation Sequencing, NGS) in order to analyze the gene subgroups encoding for immunoglobulins. A comparative study of the distribution of immunoglobulin gene subgroups present in the four libraries has revealed shifts in the B cell repertoire originating from differences in genetic background and immunological status of mice.

## Introduction

**Figure 1:**
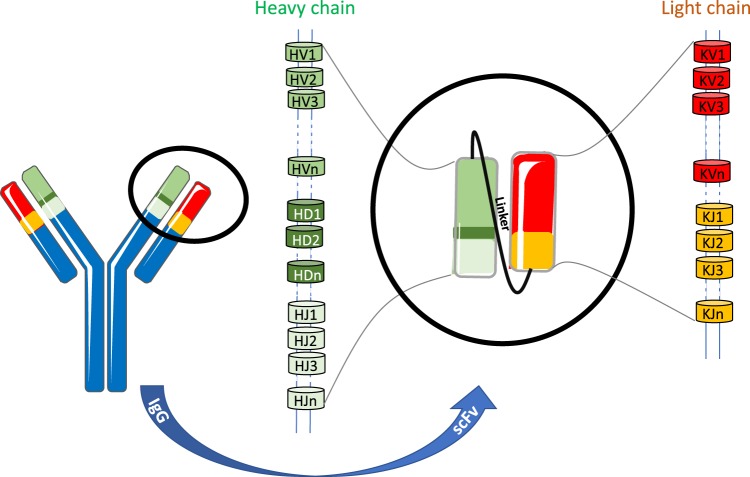
Recombination of gene segments leading to variable region diversity of immune repertoire.

The adaptive immune system is capable of producing antibodies against a large number of immunogens. This vast diversity of immunoglobulin sequences is not provided by the limited number of genes present in the genome, but by rearrangements of the germline at specific loci. In the case of B cell receptors, rearrangement of variable (V), diversity (D), and joining (J) gene segments in V-Domain creates a combinatorial diversity for the immunoglobulin heavy chain (IGH), whereas rearrangement of V and J gene segments provides a similar diversity for the lambda or kappa light chains (IGL/IGK)^1^ (Fig. 1). Additionally, at the junctions of V-D and D-J segments, a process of random deletion and addition of nucleotides creates an immense junctional diversity. Finally, somatic hypermutations focused on Complementary Determining Regions (CDR) supplement the mechanisms of immunoglobulin maturation, expanding still further the diversity and leading to affine and specific antibodies. Studies have shown that this vast diversity, as well as other characteristics of the immune repertoire, can be influenced by factors such as immunization^2,3^ or pathology, notably autoimmune diseases^4–6^.

Generation of antibody libraries is a crucial step in the attempt to study *in vivo* immune repertoires^7,8^. Care needs to be taken to ensure the coverage of a large antibody sequence diversity in order to mimic the natural B cell repertoire as close as possible. Recently, we have described an original strategy allowing to improve the library construction process and increase its diversity^9^. This strategy is based on a technological optimization relying on Rolling Circle Amplification (RCA), combined with a newly designed set of oligonucleotide primers based on a thorough analysis of the IMGT/LIGM-DB database^10^. In the present study, we have used this strategy to generate libraries form two murine inbred strains were used, namely Balb/C (healthy) and SJL/J (susceptible to autoimmune disease), together representing 4 different IgG immune repertoires: i) healthy and naïve (NB for naïve Balb/C), ii) healthy and immunized (IB for immunized Balb/C), iii) autoimmune prone and naïve (NS for naïve SJL/J), and iv) autoimmune prone and immunized (IS for immunized SJL/J)^11^. We have decidedly chosen to focus on the IgG repertoires, excluding the IgM immunoglobulins present in natural naïve immune repertoires^12^, with the aim of focusing our comparative study on the same isotype originating from the 4 populations. The libraries have been separately amplified before next-generation sequencing (NGS) by using bar-coded primers, with a unique barcode representing each of the libraries. Hence, the PCR products are pooled and the origin of each sequence in a pool of NGS data can be easily identified. We have finally conducted a comparative study of the library where we focused on the analysis of the germline gene usage frequencies in order to investigate the influence of genetic background and immunological status on the properties of the B cell repertoire.

Hence, we report here a large-scale sequence analysis of four immune repertoires incorporated in an antibody display library. Antibody sequences were analyzed and more than 750 000 gene segments were identified. The gene subgroup distribution profiles were determined according to the immunization status and the genetic background of mice, and showed significant differences. Such studies could lay the foundation to better understand immune repertoire specificities under physiopathological contexts like autoimmune diseases.

## Results

### NGS data and Library diversity

The NGS data generated 351 286 sequences specific to immunoglobulins analyzed using IMGT/HighV-QUEST. Within this population, we observed 2 915 pseudogenes (ie 0.83%), which is consistent with our previous estimations^11^, and with findings of other teams^13^. The presence of pseudogenes in our library can be explained by the potential homology of some designed primers with pseudogene sequences, despite the option of taking into account only “functional” genes in the IMGT/LIGM-DB database manipulation during the conception of our library.

**Figure 2:**
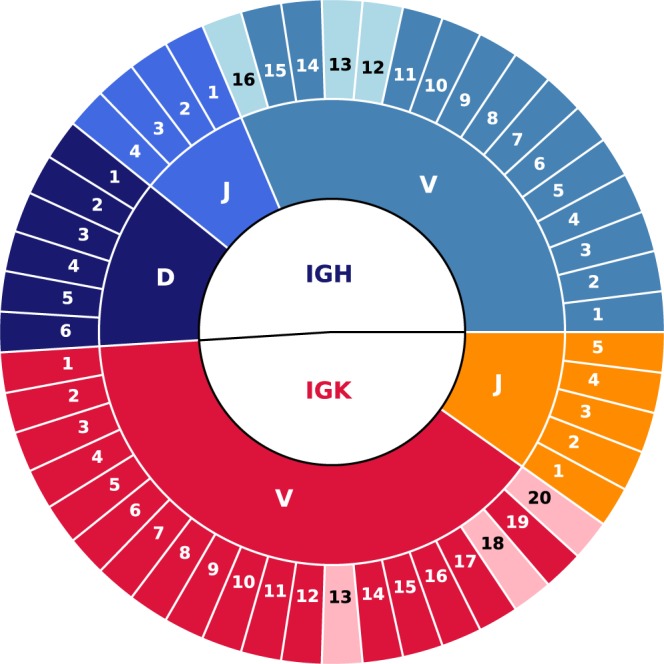
Representativity of each immunoglobulin gene subgroup in the sequenced library for κ- and γ-chains (respectively in red and blue gradients). Light slices are gene subgroups that are not represented whereas the dark ones are indeed represented.

Despite the commercial guarantees promising modal read lengths of up to 800–900 bp (GS FLX++ Technology), our sequences produced reads on average of about 520 bp. To date, obtaining high quality NGS reads of the full-length scFv remains a challenge^14^. Full-length single chain Fragment variable (scFv) sequencing is an absolute requirement for analyzing the original association of VH and VL domains, but it does not appear essential for the analysis of the gene subgroups distribution. For this reason, all of the 351 286 sequences reads -complete or not- were included in the study. These sequences gather 759 214 gene segments. According to the IMGT database, there are 16 distinct functional IGHV subgroups, 6 IGHD, and 4 IGHJ for the γ-heavy chain, 20 distinct functional IGKV, and 5 IGKJ for the κ-light chain. Data obtained by NGS suggest that a large fraction of the subgroups are indeed represented, ie 13 out of the 16 IGHV, and all the IGHD and IGHJ. Concerning the light chains, 17 out of the 20 IGKV, and all the IGKJ are represented (Fig. 2). Taken together, more than 88% of the gene subgroups expected to be present are indeed represented, confirming the large diversity of the library and a near-complete coverage. Such a conclusion was suggested in a previous study on 400 sequences and is now validated.

### Gene subgroup distribution

**Figure 3:**
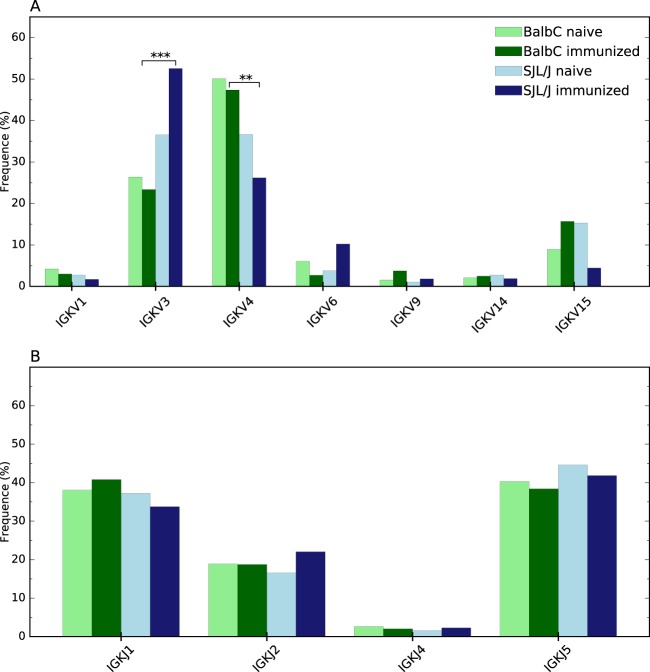
Immunoglobulin gene subgroup distribution of κ-light chain, for (**A**) IGKV and (**B**) IGKJ. Only gene subgroups with representativity >2% are illustrated (raw data are available in supplementary data). Statistical differences with p < 0.05 are represented by *, p < 0.01 by **, and p < 0.001 by ***.

**Figure 4:**
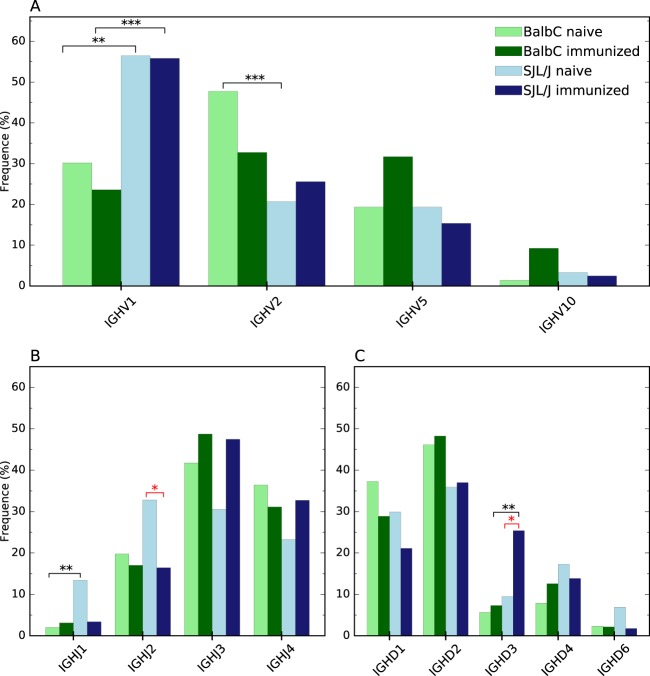
Immunoglobulin gene subgroup distribution of γ-heavy chain for (**A**) IGHV, (**B**) IGHJ and (**C**) IGHD. Only gene subgroups with representativity >2% are illustrated (raw data are available in supplementary data). Statistical differences with p < 0.05 are represented by *, p < 0.01 by **, and p < 0.001 by ***. Black stars are associated to a comparison between immunization statuses whereas red ones are associated to a comparison between genetic backgrounds of mice.

The determination of gene subgroups of the 351 286 sequences was performed and led to the identification of 759 214 gene segments. The distribution of immunoglobulin G gene subgroups for each chain was further studied in order to compare their level of representation between the 4 libraries. The purpose is to investigate whether the genetic background (healthy or autoimmune prone) and/or immunological status of mice (naïve or immunized), influence the immunoglobulin gene expression pattern. The statistical Pearson’s Chi-squared test has been used to analyze the results. The expression profiles of gene segments for each of the 4 libraries are shown in Figs 3 and 4. Distribution appears indeed to be significantly different for several gene subgroups. It is important to underline that the statistical test was performed only on subgroups for which a significant difference (p-value < 0.05) was observed by applying the test to each segment globally and to pairs of repertoires. In addition, only subgroups having a population of at least 2% for one mouse are reported (Figs 3 and 4). The entire lists of gene subgroups and their frequencies can be found as Supplementary Tables S1 to S5.

Only two gene subgroups are influenced by the effect of immunization, whereas the strain impacts seven gene subgroups. Indeed, immunization leads to a significant overexpression of IGHD3 and to an underexpression of IGHJ2, both for SJL/J mice.

Additional differences in the immunoglobulin gene expression profiles emerged between the two strains of mice with different genetic backgrounds but the same status of immunization. Concerning the naïve repertoires, the subgroup IGHV2 is significantly overexpressed in Balb/C *versus* SJL/J, whereas IGHV1 and IGHJ1 are overexpressed in naïve SJL/L *versus* Balb/C. In immunized repertoires from both strains, the subgroups IGKV3, IGHV1 and IGHD3 have a significant lower percentage of representation in Balb/C mice compared to SJL/J mice, whereas the opposite is observed for IGKV4, where the subgroup is overexpressed in Balb/C compared to SJL/J.

Our results also indicate that the observed significant differences spread out over the V, D and J segments of the γ-heavy chain, whereas for κ-light chain, the differences focus on the V segment, essentially on the more frequent gene subgoups -the least frequent subgroups being the least impacted.

## Discussion

Our library of size 2.6 × 10^9^ in size gathers 4 distinct immune repertoires (NB, IB, NS and IS), each reflecting a unique mouse^11^. It is noteworthy that a specific murine strain is generated from a rigorous selection applied over many generations, thus producing mice with identical genes. Balb/C and SJL/J are inbred mice and are considered as homozygotes for each allele. Moreover, previous works based on 38 blood samples of human volunteers suggest that the use of the germline diversity is strongly stereotyped between individuals^15^. Additionally, works by Glanville and collaborators showed that monozygotic twins -that can be correlated to inbred mice- share nearly identical naïve gene segment usage profiles. Furthermore, these studies have shown that gene segment usage is a function of the genetic origins of individuals rather than environmental factors and homeostasis^16^. Finally, Greiff *et al*. recently suggested that genetic background highly predetermines antibody repertoires independently of mouse strains, thereby validating the expandability of our results to other mice from the same strains^17^. For all of these reasons, we can expect that gene subgroups distribution is similar between individuals belonging to the same strain.

The gene subgroup distribution study shows that, within a given murine strain, immunization led to a slight shift in the B cell repertoire. It is well described that an immunization event induces a series of somatic mutations in the immunoglobulin sequences of the same gene subgroup, in order to select most affine antibodies. Rubelt and collaborators demonstrated that the choice of V(D)J segments most likely has a direct impact on antigen specificity. This group showed that significant shift in the proportion of IGV genes was observed systematically for genes belonging to the same gene subgroup^18^. By studying the gene subgroup distribution rather than focusing on the genes, the approach is more global and the conclusion is quite different, since the improvement of a specific gene will not systematically influence all gene subgroups. Clonal selection induces implicitly the overexpression of a specific gene and consequently the underexpression of other specific genes, but the impact on the gene subgroup is not systematic.

Moreover, regardless of the immunization status, we observe a difference in the immunoglobulin gene distribution profiles, depending on the murine strain. Not only the strain effect impacts more gene subgroups than the immunization effect (7 *versus* 2), but also the statistical significance is higher (p-value < 0.05 *versus* p-value < 0.01 or even < 0.001). Such differences between two distinct strains (Balb/C *versus* C57BL/6) have already been suggested by previous works^19^. In parallel, some authors demonstrated that subsets of V-segments exhibit biased representation in infection, B-cell lymphoma or even in autoimmunity^16,20–22^. The SJL/J strain is different from the Balb/C strain due to a mutation in the dysferlin protein. It has been shown that this strain shows a permissive T cell repertoire capable of recognizing multiple determinants of specific antigens, making them prone to autoimmune diseases^23,24^. However, the genetic origin of this elevated sensitivity to autoimmunity is yet to be discovered. Since our data suggest that the genetic background of mice influence their B cell repertoire, we can conclude that a specific distribution profile of immunoglobulin genes could explain the emergence of autoimmune diseases, or at least characterize them. In other words and as previously suggested by Glanville *et al*.^16^, the heritable naïve V-gene profiles may contribute to autoimmune susceptibility. Etiology of autoimmune diseases is far from being completely understood but should be maximized by the study of gene subgroup variations.

The immune system is devoted to produce affine antibodies. During the years, evolution has favored genes encoding for immunoglobulins with binding abilities, allowing the well-known affinity maturation process. For this reason, we can expect to observe a specific gene subgroup distribution in healthy strains, the most frequent gene subgroups being the most suitable for affinity maturation. We hypothesize that a pathological context such as an autoimmune disease could induce an important shift in the gene expression profile, leading to antibodies with new properties. Numerous authors suggested a link between autoimmune diseases and catalytic antibodies^25–28^. Catalytic antibodies, in contrast with conventional “binding” antibodies, are immunoglobulins endowed with catalytic properties^29–31^. They were initially elicited from a deliberate manipulation of the immune system, but they were subsequently described in specific physiopathological contexts, under which they appeared spontaneously. Hence, we hypothesize that an important shift in the gene profile due to an autoimmune disease could induce expression of genes more susceptible to generate catalytic antibodies. In this context, the well described affinity maturation process would give its place to a “catalytic maturation” process, relying on distinct mechanisms and/or distinct genes.

In conclusion, high throughput sequencing performed on our antibody library has been used to analyze the characteristics attributed to various immune repertoires. We report a relevant study of the differences in immunoglobulin gene expression between two strains of mice with different genetic backgrounds and distinct immunological status. Our data show that genetic background and, to a lesser extent the immunological status of mice, influence their B cell repertoire. Such studies are fundamental for the future development of diagnostic measures for autoimmune diseases. High throughput studies of the differences between the immunized *versus* non-immunized libraries can also provide further fundamental information on the trends of maturation of the immune repertoire post-immunization, as suggested by DeKoksy and collaborators^32^. These studies can potentially aid the methodology of engineering vaccines in order to target a particular immune response.

## Methods

### NGS sequencing

The phagemides pAK100, pAK101, pAK102 and pAK103 were independently prepared as previously described^11^. 100 µL of each phage preparation was added to 100 mL of *E. coli* bacteria, cultured overnight at 37 °C in LB medium with chloramphenicol 25 µg.mL^−1^. After a Maxiprep plasmid extraction (QIAGEN Plasmid Maxi Kit, # 12162), PCR amplifications were performed using 50 ng of template DNA, 0.5 µM of each primer, MgSO_4_ 2 mM, dNTP 200 µM and Taq Polymerase 1U (NewEngland Biolabs, # MO273S). The Forward and Reverse primers were designed according to commercial recommendations and are listed in Supplementary Table S6. The PCR thermocycler program was 95 °C for 3 min, 30 cycles (95 °C for 30 s, 65 °C for 30 s, 73 °C for 45 s), 73 °C for 7 min and 4 °C storage. Four reactions were performed (one for each library), repeated several times in order to reach required concentration of each PCR product according to the commercial instructions. PCR products were pooled, purified on PCR purification columns (QIAquick PCR Purification kit, # 28106), and submitted for GS FLX++ technology (outsource: Eurofin).

### Sequences analysis using IMGT High-VQuest

The FASTA file resulting from NGS sequencing was submitted to IMGT/HighV-QUEST (http://www.imgt.org/HighV-QUEST/login.action)^33–35^ which implements IMGT/HighV-QUEST program version 1.5.5 (June 9, 2017), IMGT/V-QUEST program version 3.4.7 (June 8, 2017) and IMGT/V-QUEST reference directory release 201728-2 (July 11, 2017). The analysis was performed with the advanced functionality “Analysis of single chain Fragment variable (scFv)”. The closest germline IMGT gene was determined for each gene segment, in order to study the distribution of each gene subgroup according to the library, ie NB, IB, NS and IS.

### Statistical analysis

The significance of the investigated differences in the distribution of immunoglobulin gene subgroups between the four considered libraries has been assessed using a randomized Pearson’s Chi-squared statistical test^36,37^.

The test was applied using scipy.stats.chi2_contingency python function^38^, which computes the chi-square statistic and p-value for the hypothesis test of independence of the observed frequencies in the provided contingency table. The expected frequencies are computed based on the marginal sums under the assumption of independence.

In addition, in order to obtain solid results, the test was applied at different levels. More in detail, we initially verified if there is a significant difference (p –value < 0.001) between subgroups within a whole fragment (Supplementary Table S7). If it was the case (all the segments except for IGKJ), we proceeded to verify the significant difference within segments between couples of repertoires (naïve Balb/C *vs* immunized Balb/C, naïve SJL/J *vs* immunized SJL/J, naïve Balb/C *vs* naïve SJL/J, immunized Balb/C *vs* immunized SJL/J, Supplementary Table S8). If it was the case, we finally applied the test on each subgroup in a repertoire-pairwise fashion (Supplementary Table S9).

Moreover, at the three levels, the test was applied by randomly selecting 100 elements for each repertoire and repeating it 1000 times. The final p-value corresponded to the median of the p-values obtained from the 1000 rounds. When the test was applied to the last two levels the Bonferroni correction for multiple comparisons was applied^39^. The test could be applied only if the subgroup counts were greater than 5, due to the limits of the test. This procedure is necessary because of the dimensions and type of the data for each segment.

## Supplementary information

Tables S1 to S9

## Data Availability

All authors declare that materials, data and associated protocols remains available to readers without undue qualifications in material transfer agreements.
