# Supplementary material for: Genetic background and immunological status influence B cell repertoire diversity in mice
Source: Sci Rep. 2019 Oct 3;9:14261. doi: 10.1038/s41598-019-50714-y (PMC6776527; doi:10.1038/s41598-019-50714-y)
Supplement: Supplementary file 1 — Tables S1 to S9 [file 41598_2019_50714_MOESM1_ESM.pdf]

Genetic background and immunological status influence B cell repertoire diversity in mice

Nancy Chaaya[a,b], Melody A Shahsavarian[a,b], Irene Maffucci[a,b], Alain Friboulet[a,b], Bernard Offmann[c], Jean-Benoist Leger[d,e], Sylvain Rousseau[d,e], Bérangère Avalle[a,b], Séverine Padiolleau-Lefevre[a,b,*]

[a]*CNRS UMR 7025, Génie Enzymatique et Cellulaire. Centre de Recherche de Royallieu. CS 60319, 60203 Compiègne Cedex, France.*
[b]*Sorbonne Universités, Université de Technologie de Compiègne, Génie Enzymatique et Cellulaire. Centre de Recherche de Royallieu. CS 60319, 60203 Compiègne Cedex, France.*
[c]*Université de Nantes, Unité Fonctionnalité et Ingénierie des Protéines (UFIP), UMR 6286 CNRS, UFR Sciences et Techniques, 2, chemin de la Houssinière, 44322 Nantes, France.*
[d]*CNRS UMR 7253, Heudiasyc ; Université de technologie de Compiègne. Centre de Recherche de Royallieu. CS 60319, 60203 Compiègne Cedex, France.*
[e]*Sorbonne Universités, Université de technologie de Compiègne, Heudiasyc. Centre de Recherche de Royallieu. CS 60319, 60203 Compiègne Cedex, France.*

SUPPORTING INFORMATION

Table S1. Representativity of IGHV gene subgroups.
Analysis of NGS data was performed using IMGT/HighV-QUEST, for each murine strain and for V(D)J segments of the γ heavy chain (A) and κ light chain (B). NB denotes the Naïve Balb/C mouse, IB to the Immunized Balb/C, NS to the Naïve SJL/J, and IS to the Immunized SJL/J one. Gene subgroups in grey correspond to the gene subgroups referenced in the IMGT database but not represented in our library. Gene subgroups having a representativity inferior to 2% for each murine strain are in italic and not illustrated in figures 3 and 4.

IGHV								
	NB		IB		NS		IS	
	Row data	(%)	Row data	(%)	Row data	(%)	Row data	(%)
**IGHV01**	**7470**	**30,2%**	**2227**	**23,6%**	**4270**	**56,5%**	**11151**	**55,8%**
**IGHV02**	**11813**	**47,8%**	3091	32,7%	**1563**	**20,7%**	5108	25,6%
IGHV03	*82*	*0,3%*	*66*	*0,7%*	*0*	*0,0%*	*32*	*0,2%*
IGHV04	*0*	*0,0%*	*4*	*0,0%*	*0*	*0,0%*	*0*	*0,0%*
IGHV05	4788	19,4%	2994	31,7%	1462	19,3%	3064	15,3%
IGHV06	*9*	*0,0%*	*3*	*0,0%*	*0*	*0,0%*	*0*	*0,0%*
IGHV07	*45*	*0,2%*	*67*	*0,7%*	*4*	*0,1%*	*16*	*0,1%*
IGHV08	*0*	*0,0%*	*0*	*0,0%*	*0*	*0,0%*	*1*	*0,0%*
IGHV09	*168*	*0,7%*	*93*	*1,0%*	*3*	*0,0%*	*0*	*0,0%*
IGHV10	344	1,4%	871	9,2%	249	3,3%	493	2,5%
IGHV11	*1*	*0,0%*	*9*	*0,1%*	*1*	*0,0%*	*0*	*0,0%*
IGHV12	*0*	*0,0%*	*0*	*0,0%*	*0*	*0,0%*	*0*	*0,0%*
IGHV13	*0*	*0,0%*	*0*	*0,0%*	*0*	*0,0%*	*0*	*0,0%*
IGHV14	*15*	*0,1%*	*15*	*0,2%*	*2*	*0,0%*	*9*	*0,0%*
IGHV15	*4*	*0,0%*	*0*	*0,0%*	*6*	*0,1%*	*100*	*0,5%*
IGHV16	*0*	*0,0%*	*0*	*0,0%*	*0*	*0,0%*	*0*	*0,0%*
TOTAL	24739	100%	9440	100%	7560	100%	19974	100%

Table S2. Representativity of IGHJ gene subgroups.

Analysis of NGS data was performed using IMGT/HighV-QUEST, for each murine strain and for V(D)J segments of the γ heavy chain (A) and κ light chain (B). NB denotes the Naïve Balb/C mouse, IB to the Immunized Balb/C, NS to the Naïve SJL/J, and IS to the Immunized SJL/J one.

IGHJ								
	NB		IB		NS		IS	
	Row data	(%)	Row data	(%)	Row data	(%)	Row data	(%)
**IGHJ1**	**501**	**2,0%**	295	3,1%	**1012**	**13,4%**	675	3,4%
**IGHJ2**	4895	19,8%	1605	17,0%	**2481**	**32,8%**	**3283**	**16,4%**
IGHJ3	10330	41,8%	4601	48,7%	2309	30,5%	9477	47,5%
IGHJ4	9012	36,4%	2939	31,1%	1757	23,2%	6537	32,7%
TOTAL	24738	100%	9440	100%	7559	100%	19972	100%

Table S3. Representativity of IGHD gene subgroups.

Analysis of NGS data was performed using IMGT/HighV-QUEST, for each murine strain and for V(D)J segments of the γ heavy chain (A) and κ light chain (B). NB denotes the Naïve Balb/C mouse, IB to the Immunized Balb/C, NS to the Naïve SJL/J, and IS to the Immunized SJL/J one.

IGHD								
	NB		IB		NS		IS	
	Row data	(%)	Row data	(%)	Row data	(%)	Row data	(%)
IGHD1	8324	37,3%	2264	28,9%	2079	29,9%	4122	21,1%
IGHD2	10306	46,1%	3786	48,3%	2494	35,9%	7219	37,0%
**IGHD3**	1259	5,6%	**573**	**7,3%**	**659**	**9,5%**	**4959**	**25,4%**
IGHD4	1757	7,9%	988	12,6%	1199	17,3%	2703	13,8%
IGHD5	175	0,8%	66	0,8%	36	0,5%	173	0,9%
IGHD6	518	2,3%	168	2,1%	480	6,9%	342	1,8%
TOTAL	22339	100%	7845	100%	6947	100%	19518	100%

Table S4. Representativity of IGKV gene subgroups.
Analysis of NGS data was performed using IMGT/HighV-QUEST, for each murine strain and for V(D)J segments of the γ heavy chain (A) and κ light chain (B). NB denotes the Naïve Balb/C mouse, IB to the Immunized Balb/C, NS to the Naïve SJL/J, and IS to the Immunized SJL/J one. Gene subgroups in grey correspond to the gene subgroups referenced in the IMGT database but not represented in our library. Gene subgroups having a representativity inferior to 2% for each murine strain are in italic and not illustrated in figures 3 and 4.

IGKV								
	NB		IB		NS		IS	
	Row data	(%)	Row data	(%)	Row data	(%)	Row data	(%)
IGKV01	3214	4,2%	1375	3,0%	911	2,7%	2237	1,7%
IGKV02	*183*	*0,2%*	*140*	*0,3%*	*215*	*0,6%*	*742*	*0,6%*
**IGKV03**	20088	26,4%	**10818**	**23,4%**	12318	36,5%	**70094**	**52,5%**
**IGKV04**	38144	50,1%	**21933**	**47,4%**	12351	36,6%	**34946**	**26,2%**
IGKV05	*206*	*0,3%*	*279*	*0,6%*	*116*	*0,3%*	*466*	*0,3%*
IGKV06	4604	6,0%	1244	2,7%	1270	3,8%	13646	10,2%
IGKV07	*18*	*0,0%*	*4*	*0,0%*	*3*	*0,0%*	*3*	*0,0%*
IGKV08	*22*	*0,0%*	*2*	*0,0%*	*10*	*0,0%*	*21*	*0,0%*
IGKV09	1198	1,6%	1724	3,7%	345	1,0%	2414	1,8%
IGKV10	*16*	*0,0%*	*12*	*0,0%*	*9*	*0,0%*	*15*	*0,0%*
IGKV11	*1*	*0,0%*	*0*	*0,0%*	*0*	*0,0%*	*0*	*0,0%*
IGKV12	*46*	*0,1%*	*11*	*0,0%*	*11*	*0,0%*	*97*	*0,1%*
IGKV13	*0*	*0,0%*	*0*	*0,0%*	*0*	*0,0%*	*0*	*0,0%*
IGKV14	1579	2,1%	1147	2,5%	909	2,7%	2484	1,9%
IGKV15	6794	8,9%	7262	15,7%	5147	15,3%	5924	4,4%
IGKV16	*24*	*0,0%*	*346*	*0,7%*	*89*	*0,3%*	*314*	*0,2%*
IGKV17	*2*	*0,0%*	*1*	*0,0%*	*0*	*0,0%*	*6*	*0,0%*
IGKV18	*0*	*0,0%*	*0*	*0,0%*	*0*	*0,0%*	*0*	*0,0%*
IGKV19	*3*	*0,0%*	*9*	*0,0%*	*1*	*0,0%*	*10*	*0,0%*
IGKV20	*0*	*0,0%*	*0*	*0,0%*	*0*	*0,0%*	*0*	*0,0%*
TOTAL	76142	100%	46307	100%	33705	100%	133419	100%

Table S5. Representativity of IGKJ gene subgroups.
Analysis of NGS data was performed using IMGT/HighV-QUEST, for each murine strain and for V(D)J segments of the γ heavy chain (A) and κ light chain (B). NB denotes the Naïve Balb/C mouse, IB to the Immunized Balb/C, NS to the Naïve SJL/J, and IS to the Immunized SJL/J one.

IGKJ								
	NB		IB		NS		IS	
	Row data	(%)	Row data	(%)	Row data	(%)	Row data	(%)
IGKJ1	28986	38,1%	18887	40,8%	12547	37,2%	45050	33,8%
IGKJ2	14382	18,9%	8677	18,7%	5579	16,6%	29420	22,1%
IGKJ3	47	0,1%	20	0,0%	11	0,0%	69	0,1%
IGKJ4	2019	2,7%	938	2,0%	533	1,6%	3041	2,3%
IGKJ5	30705	40,3%	17785	38,4%	15035	44,6%	55839	41,9%
TOTAL	76139	100%	46307	100%	33705	100%	133419	100%

Table S6. Primers set.

Primers targeting the vector used to amplify genes adapted for NGS sequencing, according to the library (NB for Naïve and Balb/C; IB for Immunized Balb/C; NS for Naïve SJL/J, and IS for Immunized SJL/J mice). The blue characters correspond to the A-key, red ones to the key, green to the MID, and bold characters hybridize on the vector. A-key and key sequences were imposed by Eurofins. The MID sequences allow to identify the library origin.

**Primer**	**Sequence (5'… 3')**	**GC (%)**
Forprimer-BN	CGTATCGCCTCCCTCGCGCCATCAGACGAGTGCGT**TACGGCAGCCGCTGGATTGT**	63,64
Revprimer-BN	CTATGCGCCTTGCCAGCCCGCTCAGACGAGTGCGT**AGCTTCTGCTCGAATTCGGC**	61,82
Forprimer-BI	CGTATCGCCTCCCTCGCGCCATCAGCGTGTCTCTA**TACGGCAGCCGCTGGATTGT**	61,82
Revprimer-BI	CTATGCGCCTTGCCAGCCCGCTCAGCGTGTCTCTA**AGCTTCTGCTCGAATTCGGC**	60,00
Forprimer-SN	CGTATCGCCTCCCTCGCGCCATCAGTGATACGTCT**TACGGCAGCCGCTGGATTGT**	60,00
Revprimer-SN	CTATGCGCCTTGCCAGCCCGCTCAGTGATACGTCT**AGCTTCTGCTCGAATTCGGC**	58,18
Forprimer-SI	CGTATCGCCTCCCTCGCGCCATCAGTCTAGCGACT**TACGGCAGCCGCTGGATTGT**	61,82
Revprimer-SI	CTATGCGCCTTGCCAGCCCGCTCAGTCTAGCGACT**AGCTTCTGCTCGAATTCGGC**	60,00

Table S7. Median p-values for each gene segment.

Median p-values resulting from the repetition 1000 of times of the Chi Square test on 100-element random samples for each segment.

**Segment**	**Median p-value**
IGHV	$6.4 \cdot 10^{-8}$
IGHJ	$1.4 \cdot 10^{-5}$
IGHD	$1.6 \cdot 10^{-5}$
IGKV	$2.8 \cdot 10^{-5}$
IGKJ	0.32

Table S8. Median p-value for each segment in a repertoire-pairwise fashion.

Median p-values resulting from the 1000 times repetition of the Chi Square test on 100-element random samples for each segment in a repertoire-pairwise fashion (naïve Balb/C *vs* immunized Balb/C, naïve SJL/J *vs* immunized SJL/J, naïve Balb/C *vs* naïve SJL/J, immunized Balb/C *vs* immunized SJL/J). The Bonferroni's correction has been applied. The test has not been applied on the IGKJ segment, which didn't show a global significant difference (Table S7).

**Segment**	**Compared repertoires**	**Corrected median p-value**
IGHV	naïve Balb/C *vs* immunized Balb/C	0.028
	naïve SJL/J *vs* immunized SJL/J	1.3
	naïve Balb/C *vs* naïve SJL/J	$6.3 \cdot 10^{-4}$
	immunized Balb/C *vs* immunized SJL/J	$1.4 \cdot 10^{-4}$
IGHJ	naïve Balb/C *vs* immunized Balb/C	1.3
	naïve SJL/J *vs* immunized SJL/J	0.0014
	naïve Balb/C *vs* naïve SJL/J	0.0017
	immunized Balb/C *vs* immunized SJL/J	1.9

IGHD	naïve Balb/C *vs* immunized Balb/C	1.0
	naïve SJL/J *vs* immunized SJL/J	0.025
	naïve Balb/C *vs* naïve SJL/J	0.088
	immunized Balb/C *vs* immunized SJL/J	0.015
IGKV	naïve Balb/C *vs* immunized Balb/C	0.69
	naïve SJL/J v*s* immunized SJL/J	0.027
	naïve Balb/C *vs* naïve SJL/J	0.37
	immunized Balb/C *vs* immunized SJL/J	$7.0\cdot10^{-5}$

Table S9. Median p-value for gene subgroup in a repertoire-pairwise fashion.
Median p-values resulting from the 1000 times repetition of the Chi Square test on 100-element random samples for each subgroup in a repertoire-pairwise fashion (naïve Balb/C *vs* immunized Balb/C, naïve SJL/J *vs* immunized SJL/J, naïve Balb/C *vs* naïve SJL/J, immunized Balb/C *vs* immunized SJL/J). The Bonferroni's correction has been applied. The test has not been applied on the IGKJ segment and on couples of repertoires having a p-value > 0.05 (Table S7, S8). Only subgroups with a count > 5 were submitted to the test. The significant level is indicated between brackets.

Segment	Subgroup	Compared repertoires	Corrected median p-value
IGHV	1	naïve Balb/C *vs* immunized Balb/C	0.35
		naïve Balb/C *vs* naïve SJL/J	$3.2\cdot10^{-3}$ (**)
		immunized Balb/C *vs* immunized SJL/J	$8.2\cdot10^{-5}$ (***)
	2	naïve Balb/C *vs* immunized Balb/C	0.085
		naïve Balb/C *vs* naïve SJL/J	$8.4\cdot10^{-4}$ (***)
		immunized Balb/C *vs* immunized SJL/J	4.9
	3	naïve Balb/C *vs* immunized Balb/C	3.0
		immunized Balb/C *vs* immunized SJL/J	15
	5	naïve Balb/C *vs* immunized Balb/C	0.24
		naïve Balb/C *vs* naïve SJL/J	6.5
		immunized Balb/C *vs* immunized SJL/J	0.15
	7	naïve Balb/C *vs* immunized Balb/C	5.0
		immunized Balb/C *vs* immunized SJL/J	17
	9	naïve Balb/C *vs* immunized Balb/C	6.0
	10	naïve Balb/C *vs* immunized Balb/C	0.17
		naïve Balb/C *vs* naïve SJL/J	7.4
		immunized Balb/C *vs* immunized SJL/J	1.5
	14	naïve Balb/C *vs* immunized Balb/C	8.0
		immunized Balb/C *vs* immunized SJL/J	19
IGHJ	1	naïve Balb/C *vs* naïve SJL/J	$6.4\cdot10^{-3}$ (**)
		naïve SJL/J *vs* immunized SJL/J	0.095
	2	naïve Balb/C *vs* naïve SJL/J	0.10
		naïve SJL/J *vs* immunized SJL/J	0.050 (*)
	3	naïve Balb/C *vs* naïve SJL/J	0.44

		naïve SJL/J *vs* immunized SJL/J	1.5
	4	naïve Balb/C *vs* naïve SJL/J	0.27
		naïve SJL/J *vs* immunized SJL/J	1.6
IGHD	1	naïve SJL/J *vs* immunized SJL/J	0.20
		immunized Balb/C *vs* immunized SJL/J	1.8
	2	naïve SJL/J *vs* immunized SJL/J	1.1
		immunized Balb/C *vs* immunized SJL/J	1.2
	3	naïve SJL/J *vs* immunized SJL/J	0.019 (*)
		immunized Balb/C *vs* immunized SJL/J	$7.0 \cdot 10^{-3}$ (**)
	4	naïve SJL/J *vs* immunized SJL/J	2.2
		immunized Balb/C *vs* immunized SJL/J	6.7
	5	naïve SJL/J *vs* immunized SJL/J	5.0
		immunized Balb/C *vs* immunized SJL/J	1.1
	6	naïve SJL/J *vs* immunized SJL/J	0.78
		immunized Balb/C *vs* immunized SJL/J	12
IGKV	1	naïve SJL/J *vs* immunized SJL/J	10
		immunized Balb/C *vs* immunized SJL/J	0.7
	2	naïve SJL/J *vs* immunized SJL/J	15
		immunized Balb/C *vs* immunized SJL/J	2
	3	naïve SJL/J *vs* immunized SJL/J	0.5
		immunized Balb/C *vs* immunized SJL/J	$1.4 \cdot 10^{-4}$ (***)
	4	naïve SJL/J *vs* immunized SJL/J	2.3
		immunized Balb/C *vs* immunized SJL/J	$9.3 \cdot 10^{-3}$ (**)
	5	naïve SJL/J *vs* immunized SJL/J	18
		immunized Balb/C *vs* immunized SJL/J	5
	6	naïve SJL/J *vs* immunized SJL/J	2.4
		immunized Balb/C *vs* immunized SJL/J	0.31
	8	naïve SJL/J *vs* immunized SJL/J	20
	9	naïve SJL/J *vs* immunized SJL/J	21
		immunized Balb/C *vs* immunized SJL/J	4.3
	10	naïve SJL/J *vs* immunized SJL/J	22
		immunized Balb/C *vs* immunized SJL/J	8
	12	naïve SJL/J *vs* immunized SJL/J	23
		immunized Balb/C *vs* immunized SJL/J	9
	14	naïve SJL/J *vs* immunized SJL/J	24
		immunized Balb/C *vs* immunized SJL/J	10
	15	naïve SJL/J *vs* immunized SJL/J	0.4
		immunized Balb/C *vs* immunized SJL/J	0.2
	16	naïve SJL/J *vs* immunized SJL/J	26
		immunized Balb/C *vs* immunized SJL/J	12
	19	immunized Balb/C *vs* immunized SJL/J	13
